# m^6^A RNA Methylation Regulator YTHDF1 Correlated With Immune Microenvironment Predicts Clinical Outcomes and Therapeutic Efficacy in Breast Cancer

**DOI:** 10.3389/fmed.2021.667543

**Published:** 2021-08-09

**Authors:** Ying Hu, Qinwen Pan, Minghao Wang, Xiang Ai, Yuzhao Yan, Yuan Tian, Yuting Jing, Peng Tang, Jun Jiang

**Affiliations:** Department of Breast Surgery, First Affiliated Hospital of Army Military Medical University, Chongqing, China

**Keywords:** N6-methyladenosine, regulators, breast cancer, immune microenvironment, immunotherapy, somatic mutation

## Abstract

**Objective:** Increasing evidence highlights the roles of N^6^-methyladenosine (m^6^A) and its regulators in oncogenesis. Herein, this study observed the associations of m^6^A regulators with breast cancer.

**Methods:** RNA-seq profiles of breast cancer were retrieved from the Cancer Genome Atlas (TCGA) database. The expression of m^6^A regulators was analyzed in tumor and normal tissues. Their expression correlations were analyzed by Spearson test. Overall survival (OS) analysis of these regulators was then presented. Gene set enrichment analysis (GSEA) was performed in high and low YTHDF1 expression groups. The correlations of YTHDF1 expression with immune cells and tumor mutation burden (TMB) were calculated in breast cancer samples. Somatic variation was assessed in high and low YTHDF1 expression groups.

**Results:** Most of m^6^A regulators were abnormally expressed in breast cancer compared to normal tissues. At the mRNA levels, there were closely relationships between them. Among them, YTHDF1 up-regulation was significantly related to undesirable prognosis (*p* = 0.025). GSEA results showed that high YTHDF1 expression was associated with cancer-related pathways. Furthermore, YTHDF1 expression was significantly correlated with T cells CD4 memory activated, NK cells activated, monocytes, and macrophages. There were higher TMB scores in YTHDF1 up-regulation group than its down-regulation group. Missense mutation and non-sense mutation were the most frequent mutation types.

**Conclusion:** Our findings suggested that dysregulated m^6^A regulator YTHDF1 was predictive of survival outcomes as well as response to immunotherapy of breast cancer, and were closely related to immune microenvironment.

## Introduction

The incidence of breast cancer continues to rise globally (1). It represents the most mortal among the female population (2). This malignancy is heterogeneous on clinical, molecular behaviors as well as response to therapies (3). This management is multidisciplinary, including locoregional (surgery or radiotherapy) as well as systemic therapies (4). At present, advanced breast cancer with distant metastasis is incurable. Bone, lung, liver, and brain are common metastatic sites (5). Individualized therapy is future therapeutic goal for breast cancer. Thus, it is vital to elucidate the mechanisms of breast cancer initiation as well as progression.

m^6^A is the most abundant modification in eukaryotic mRNAs, occupying 0.1–0.4% of the total adenine residues (6). The m^6^A modification takes on varied biological functions (7) like RNA splicing, RNA stabilities, nuclear export as well as translation (8). This process is involved in three kinds of m^6^A regulators, called as “writers,” “erasers,” and “readers,” containing “writers” (METTL3, METTL14, WTAP, RBM15, KIAA1429, ZC3H13), “readers” (YTHDC1, YTHDC2, YTHDF1, YTHDF2, HNRNP), as well as “erasers” (FTO, ALKBH5). “Writers” are responsible for catalyzing the formation of m^6^A. “Readers” are charge of decoding m^6^A methylation as well as producing functional signals. “Erasers” can remove the methyl code from targeted mRNAs. The m^6^A modification participates in carcinogenesis through regulating RNA production as well as metabolism. For instance, Niu et al. (9) demonstrated that FTO promoted breast tumor progress by inhibition of BNIP3. As in the study of Cai et al. (10), METTL3 up-regulation accelerates breast cancer cellular proliferation. Recent studies have emphasized the key implications of immune microenvironment in breast cancer progression as well as response to immunotherapy (11). Zhang et al. (12) reported that m^6^A modification was involved in tumor immune microenvironment formation. ALKBH5 m^6^A reader could regulate the response to anti-PD-1 immunotherapy through inhibiting the accumulation of tumor-infiltrating immune cells (13). Hence, evaluation of m^6^A regulators in individual breast cancer may enhance our understanding about characteristics of tumor immune microenvironment as well as improve the therapeutic efficacy of immunotherapies. In this study, we evaluated the expression patterns of m^6^A regulators and their correlations with survival outcomes, immune microenvironment, response to immunotherapy as well as somatic mutation in breast cancer.

## Materials and Methods

### Data Sourcse

From TCGA database (http://cancergenome.nih.gov/), this study collected RNA-seq profiles of breast cancer subjects on January 15, 2021. Meanwhile, the matched clinical information was also retrieved. After removing samples with survival time of 0, 902 samples including Basal, Her2, LumA, and LumB subtypes were retained for further analysis (Table 1). The microarray expression profiling of 17 normal breast tissues and 104 breast cancer tissues were retrieved from the GSE42568 dataset of the Gene Expression Omnibus (GEO; https://www.ncbi.nlm.nih.gov/gds/) repository. Also, expression profiles and follow-up information of 266 breast cancer patients were retrieved from the GSE21653 dataset.

**Table 1:** Clinicopathological characteristics of breast cancer patients in this study.

**Characteristics**	**Groups**	**Number**
Age	<65	641
	≥65	261
T	T1	232
	T2	534
	T3	103
	T4	33
*N*	N0	443
	N1	301
	N2	102
	N3	56
M	M0	886
	M1	16
Stage	Stage I	156
	Stage II	528
	Stage III	202
	Stage IV	16

### m^6^A Regulators

Totally, this study gathered 13 m^6^A regulators including five writers (METTL3, METTL14, WTAP, RBM15, ZC3H13, KIAA1429), five readers (YTHDC1, YTHDC2, YTHDF1, YTHDF2, HNRNPC), two erasers (FTO, ALKBH5). The expressions of these m^6^A regulators were assessed in breast cancer and normal tissue samples by Wilcoxon test. *P*-value was corrected with Bonferroni method. Spearson correlation analysis was presented between these regulators at the mRNA levels. |r| > 0.5 indicated a significant correlation.

### Survival Analysis

Samples with survival status of zero were removed. Overall survival (OS) is defined as the time from the date of diagnosis to death due to any cause. The patients were classified into high and low expression of each m^6^A regulator groups based on the median value of its expression. OS analyses were carried out between groups through univariate cox regression analysis. For each regulator, this study calculated *p*-value, hazard ratio (HR) as well as 95% confidence interval (CI). Regulators with *p*-value <0.05 and HR >1 were risk factors of breast cancer prognosis. Meanwhile, those with *p*-value <0.05 and HR <1 were protective factors.

### GSEA

Enrichment analysis was presented with GSEA software (14). Here, breast cancer subjects were separated into high and low YTHDF1 expression groups based on the media value of YTHDF1. Thousand gene set permutations were presented for each analysis. The expression level of YTHDF1 was considered a phenotype. According to the normalized enrichment score (NES), normalized *p*-value and false discovery rate (FDR), the enrichment pathways of each phenotype were classified. The absolute value of NES ≥1.0, normalized *p*-value ≤ 0.05 and FDR ≤ 0.25 were confirmed as meaningful gene sets.

### Cell Culture and Transfection

Human breast cancer cells MCF-7 (ATCC, USA) were grown in DMEM (Thermo, USA) containing 10% FBS in an incubator with 5% CO_2_ at 37°C. MCF-7 cells were seeded in 6-well plates. The culture medium was changed 1 day before transfection, and when the cells reached 70–90% of the growth density, the cells were transfected with synthetic siRNAs (si-YTHDF1; GenePharma, Suzhou, China) through the Lipofectamine^™^2000 Transfection Kit (Invitrogen, USA). Simultaneously, non-interfering siRNA was transfected as a negative control. The transfection process strictly followed the instructions of the kit, and the cells transfected for 48 h were collected for next research.

### Western Blot

Transfected cells were lysed on ice with cell lysate to extract total protein. The BCA method was used for protein quantification. The proteins were separated by SDS-PAGE gel electrophoresis, and transferred to PVDF membranes at a constant voltage of 80 V. After blocking with 5% skimmed milk powder TBST at room temperature for 2 h, the membranes were incubated with YTHDF1 (1/500; ab230330; Abcam), Axin2 (1:1000; ab32197; Abcam), c-myc (1:5000; ab152146; Abcam), β-catenin (1:10000; ab81305; Abcam), cyclin D1 (1:10000; ab134175; Abcam), and β-actin (1:5000; ab179467; Abcam) overnight at 4°C. After washing, the membranes were incubated with secondary antibodies at room temperature for 1 h. The color was developed by chemiluminescence, and the gel imaging system was used to analyze images. The gray value of the bands was measured.

### Somatic Variation

Somatic variant data of breast cancer that were stored in the mutation annotation format (MAF) were obtained from TCGA database. According to VarScan2 variant aggregation as well as masking data, somatic variation analyses were presented through “maftools” package (15). TMB score was determined for each patient as follows: (total mutation/total covered bases).

### CIBERSORT

The CIBERSORT algorithm was employed for estimating the fractions of 22 phenotypes of immune cells in each specimen based on gene expression profiles (16). LM22 leukocyte gene signature matrix was utilized in conjunction. Specimens with *p*-value < 0.05 were retained. For each specimen, the sum of the estimated fractions of immune cells was equal to 1. The enriched scores of each immune cell were compared in high and low YTHDF1 expression groups by Wilcoxon test.

### TMB

TMB is defined as the total number of somatic mutations per Mb base in the coding region of an exon, which is an emerging biomarker for judging the efficacy of tumor immunotherapy (17). The greater the TMB score, the better the therapy efficacy. TMB is calculated as the total number of somatic mutations/the size of the target area, and the unit is mutations/Mb. The somatic mutation data were in MAF format. In this study, the somatic mutation data processed by vanscan software were downloaded from TCGA. The “maftools” package was applied to calculate the TMB score of each sample. The difference in TMB scores between high and low YTHDF1 expression groups was assessed by Wilcoxon test.

### Differential Expression Analysis

Differentially expressed genes (DEGs) were screened between high and low YTHDF1 expression groups *via* the limma package. The screening thresholds were as follows: |log2 fold change (FC)| > 1 and adjusted *p*-value <0.05.

### Functional Enrichment Analysis

The “clusterProfiler” package was applied to present Gene Ontology (GO) and Kyoto Encyclopedia of Genes and Genomes (KEGG) pathways enrichment analysis based on YTHDF1-related DEGs (18). Terms with adjusted *p*-value < 0.05 were significantly enriched.

### Connectivity Map

CMap database (https://clue.io/) was employed for exploring candidate chemical compounds against breast cancer (19). Based on a list of DEGs, this study searched for the compounds through this database. The CMap connectivity score (range: −1 to 1) was indicative of the specificity degree related to the DEGs. The connectivity score of a compound tended to −1, suggesting that it negatively correlated with the DEGs. On the contrary, the connectivity score of a compound closer to 1 implied that it exhibited positive correlations with the DEGs. Here, the compounds with |connectivity score| ≥90 were candidate chemical agents.

### Scratch Test

The cells were seeded in a 6-well plate and placed in a CO_2_ cell incubator. After the cells were overgrown, a sterile toothpick was used to make a vertical mark of uniform thickness in each hole. After taking pictures of the initial state of the scratches at 0 h under an inverted microscope, the samples were put back into the incubator. After 24 h, the scratch state was photographed again. ImageJ software was used to calculate the scratch area. Relative migration rate = (0 h scratch area-24 h scratch area)/0 h scratch area.

### Transwell

The cells were added to the Matrigel-coated Transwell chamber (1 × 10^5^ cells/well). A medium containing 10% FBS was added to the well in the lower layer of each chamber. After culturing for 24 h, the chamber was removed. After staining with crystal violet, a cotton swab was used to gently wipe away the non-invasive cells in the upper chamber. The invasive cells were observed under an inverted microscope. Then, ImageJ software was applied to calculate the number of invasive cells.

### Statistical Analysis

All statistical analysis was carried out through the packages of R software (http://www.r-project.org/) or Graphpad Prism. Student's *t*-test, Wilcoxon test or ANOVA test was applied for comparisons between groups. *P* < 0.05 indicated statistical significance.

## Results

### Expression Patterns and Correlations of 13 m^6^A Regulators in Breast Cancer

**Figure 1:**
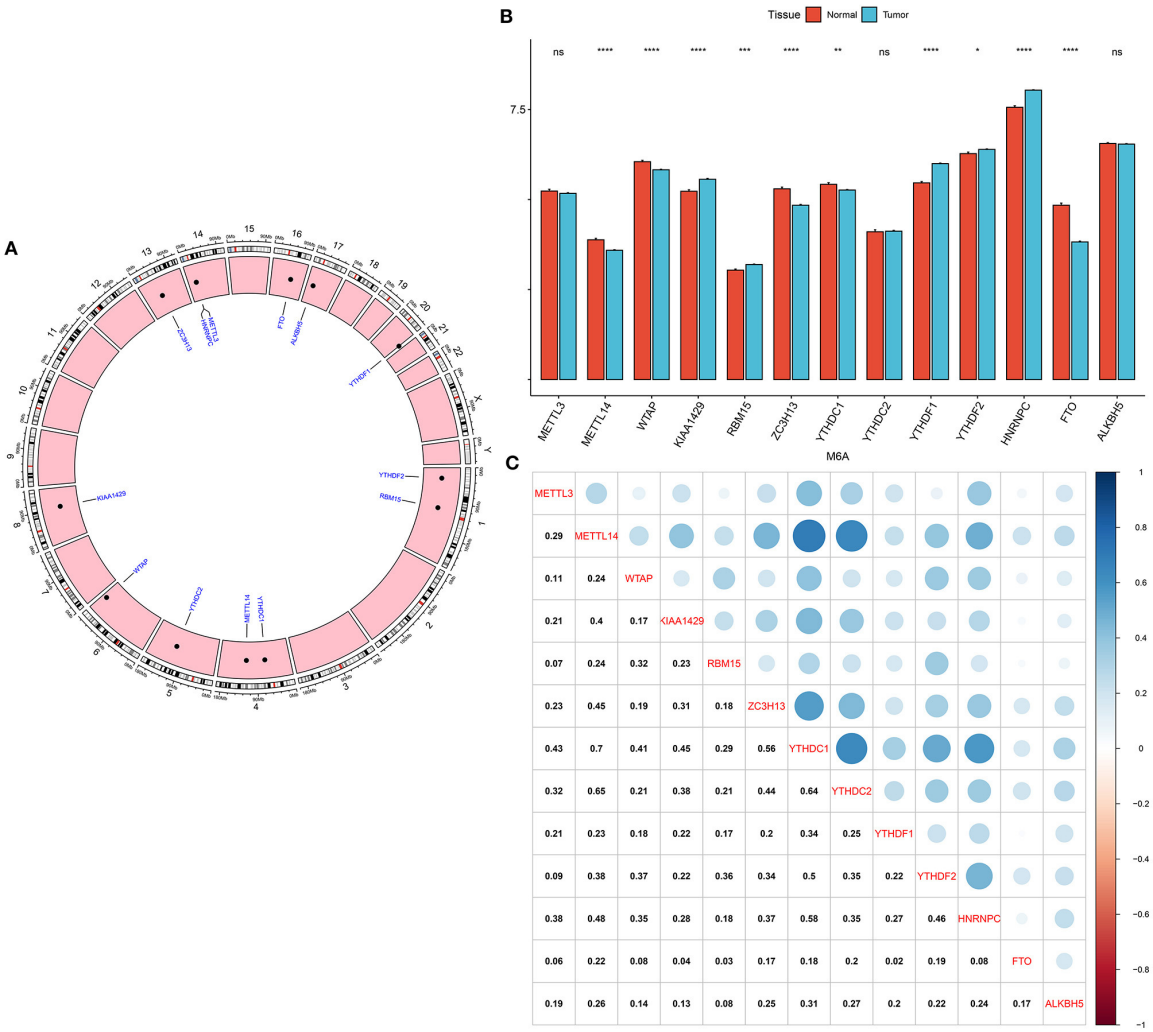
Expression patterns and correlations of 13 m^6^A regulators in breast cancer from TCGA database. **(A)** Genomic locations of the regulators. **(B)** Bar diagram for the expression of regulators in breast cancer and normal tissues. **(C)** Assessment of correlations between 13 m^6^A regulators at the mRNA levels. Ns, not significant; ^*^p < 0.05; ^**^p < 0.01; ^****^p < 0.0001.

Genomic locations for 13 m^6^A regulatory genes were displayed in Figure 1A, as follows: METTL3 (chr14: 21498133-21511342), METTL14 (chr4: 118685392-118715433), WTAP (chr6: 159725585-159756319), KIAA1429 (chr8: 94487689-94553529), RBM15 (chr1: 110338506-110346681), ZC3H13 (chr13: 45954465-46052759), YTHDC1 (chr4: 68310387-68350090), YTHDC2 (chr5: 113513694-113595285), YTHDF1 (chr20: 63195429-63216139), YTHDF2 (chr1: 28736621-28769775), HNRNPC (chr14: 21209136-21269494), FTO (chr16: 53701692-54158512) and ALKBH5 (chr17: 18183078-18209954). The expression of these regulators was compared in breast cancer and normal tissues. We found that METTL14 (adjusted *p*-value = 9.10e-08), WTAP (adjusted *p*-value = 2.86e-07), KIAA1429 (adjusted *p*-value = 2.33e-06), RBM15 (adjusted *p*-value = 1.52e-03), ZC3H13 (adjusted *p*-value = 5.56e-12), YTHDC1 (adjusted *p*-value = 1.68e-03), YTHDF1 (adjusted *p*-value = 4.72e-27), YTHDF2 (adjusted *p*-value = 4.72e-02), HNRNPC (adjusted *p*-value = 1.60e-24), and FTO (adjusted *p*-value = 2.62e-33) were significantly abnormally expressed in breast cancer compared to normal tissues (Figure 1B). However, no significant differences in METTL3, YTHDC2, and ALKBH5 expression were found between tumor and normal samples. The correlations between 13 m^6^A regulators were assessed at the mRNA levels among breast cancer specimens. Our data showed that METTL14 had a strong correlation to YTHDC1 (*r* = 0.7) and YTHDC2 (*r* = 0.65), as displayed in Figure 1C. Meanwhile, YTHDC1 was strongly associated with YTHDF1 (*r* = 0.64), and moderately correlated to HNRNPC (*r* = 0.58).

### YTHDF1 Expression Is Correlated to Breast Cancer Patients' Survival

**Figure 2:**
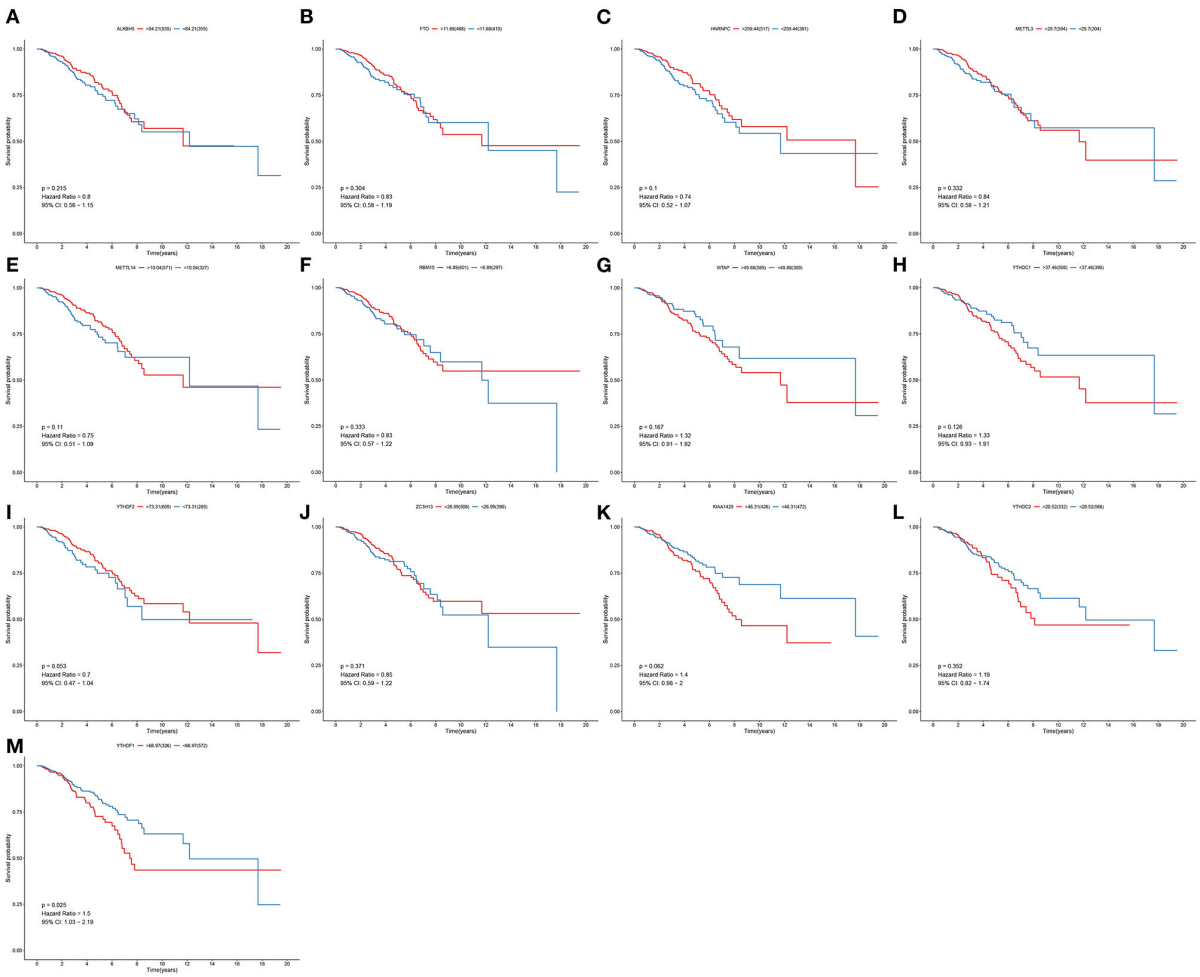
Correlation between 13 m^6^A regulators and breast cancer patients' survival. OS of **(A)** ALKBH5; **(B)** FTO; **(C)** HNRNPC; **(D)** METTL3; **(E)** METTL14; **(F)** RBM15; **(G)** WTAP; **(H)** YTHDC1; **(I)** YTHDF2; **(J)** ZC3H13; **(K)** KIAA1429; **(L)** YTHDC2; **(M)** YTHDF1 expression for breast cancer patients.

To determine the clinical implications of 13 m^6^A regulatory mRNAs in breast cancer, this study observed the correlations between the expression of 13 m^6^A regulatory mRNAs and patients' clinical outcomes. Our results showed that the expression of ALKBH5, FTO, HNRNPC, METTL3, METTL14, RBM15, WTAP, YTHDC1, YTHDF2, ZC3H13, KIAA1429, and YTHDC2 were all not associated with patients' survival (Figures 2A–L). Only YTHDF1 expression exhibited a significant correlation to subjects' prognosis (Figure 2M). Its expression was a risk factor for breast cancer (*p* = 0.025; HR: 1.5; 95% CI: 1.03–2.19). Subjects with high YTHDF1 expression often experienced undesirable survival outcomes than those with its low expression.

### Verification of Expression and Prognosis of YTHDF1 in Breast Cancer

**Figure 3:**
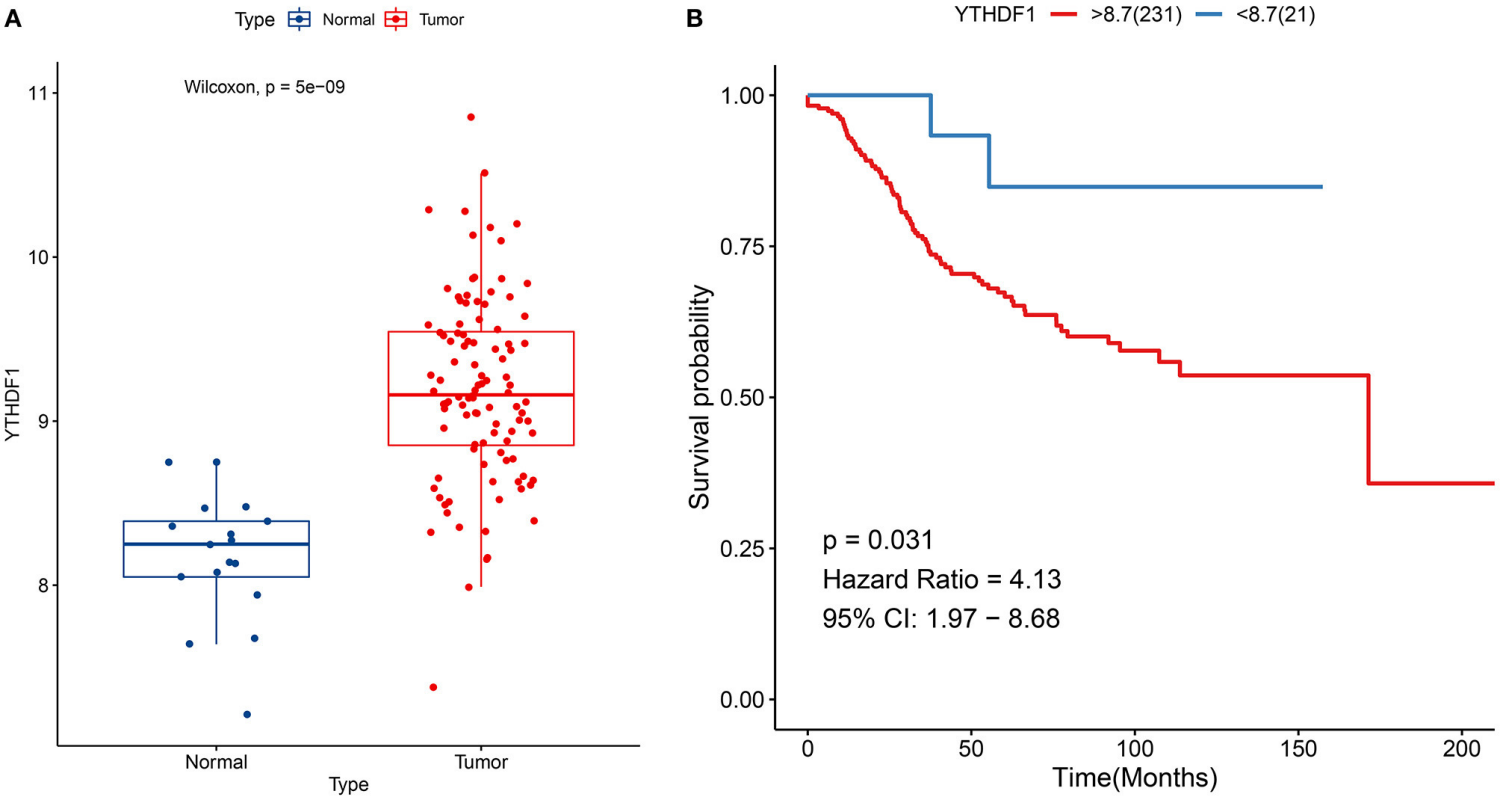
Verification of expression and prognosis of YTHDF1 in breast cancer. **(A)** Box plots for YTHDF1 expression in the GSE42568 dataset. **(B)** Survival analysis of breast cancer with high and low YTHDF1 expression in the GSE21653 dataset.

YTHDF1 up-regulation was confirmed in breast cancer in the GSE42568 dataset (*p* = 5e-09; Figure 3A). Also, its up-regulation was in relation to unfavorable survival outcomes of subjects in the GSE21653 dataset (*p* = 0.031; Figure 3B).

### Enriched Pathways in High and Low YTHDF1 Expression Groups

**Figure 4:**
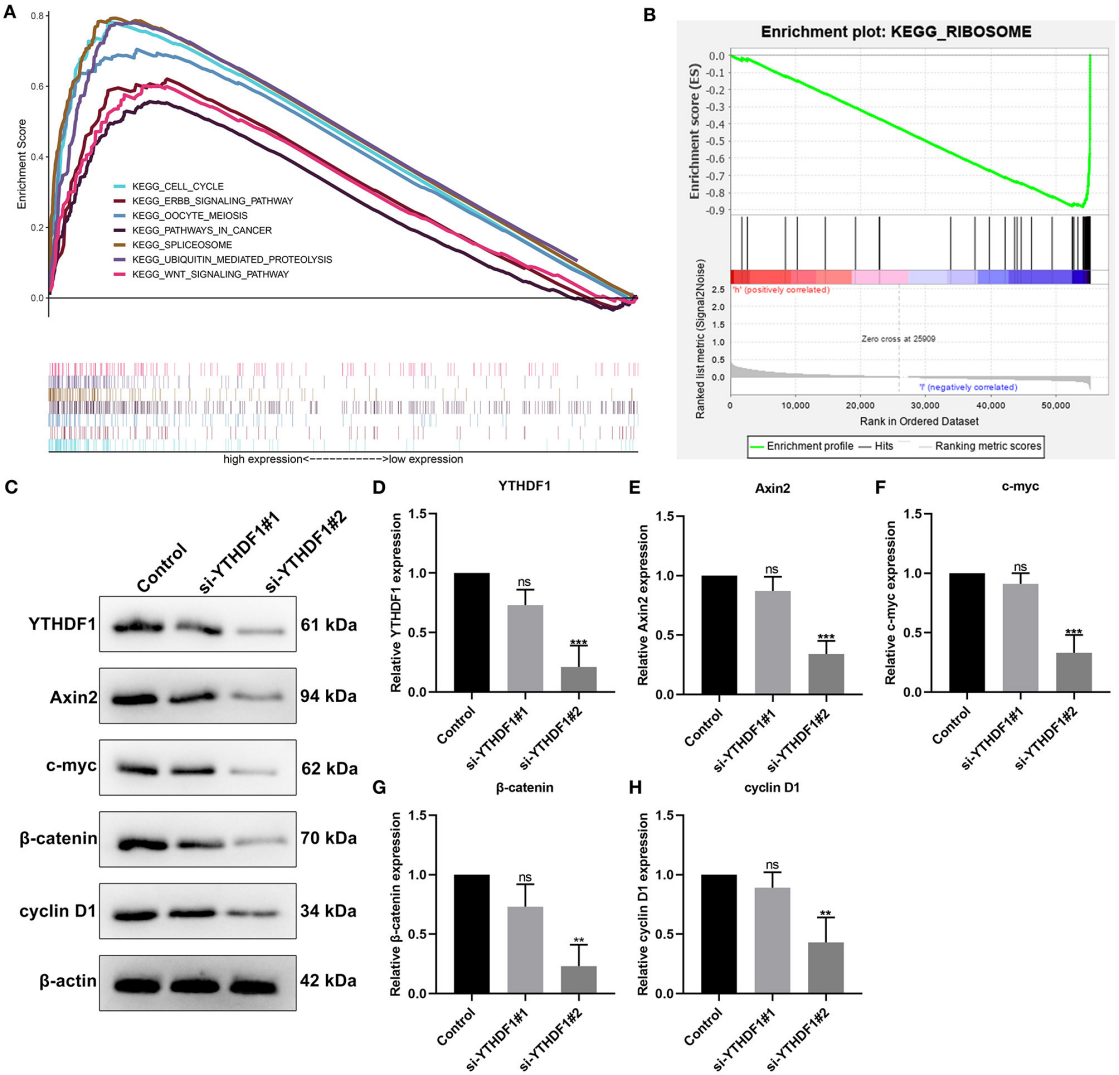
Enriched pathways in high and low YTHDF1 expression groups. **(A)** Enriched pathways in high expression group. **(B)** An enriched pathway in low expression group. **(C)** Western blot for detecting the expressions of **(D)** YTHDF1, **(E)** Axin2, **(F)** c-myc, **(G)** β-catenin and **(H)** cyclin D1 in MCF-7 cells transfected with si-YTHDF1. Ns, not significant; ^**^p < 0.01; ^***^p < 0.001.

Then, we evaluated the enriched signaling pathways in high and low YTHDF1 expression groups. We found that cell cycle, ERBB signaling pathway, oocyte meiosis, pathways in cancer, spliceosome, ubiquitin mediated proteolysis, and WNT signaling pathway were distinctly enriched in high YTHDF1 expression group (Figure 4A). Meanwhile, ribosome was significantly enriched in low YTHDF1 expression group (Figure 4B). To verify whether YTHDF1 altered WNT pathway activation, YTHDF1 was successfully silenced by siRNA in MCF-7 cells (Figures 4C,D). Our data showed that YTHDF1 knockdown distinctly lowered the expression of Axin2, c-myc, β-catenin, and cyclin D1 in MCF-7 cells, confirming that YTHDF1 participated in the activation of WNT pathway in breast cancer (Figures 4E–H).

### Correlation Between YTHDF1 Expression and Tumor Immune Microenvironment and Response to Immunotherapy

**Figure 5:**
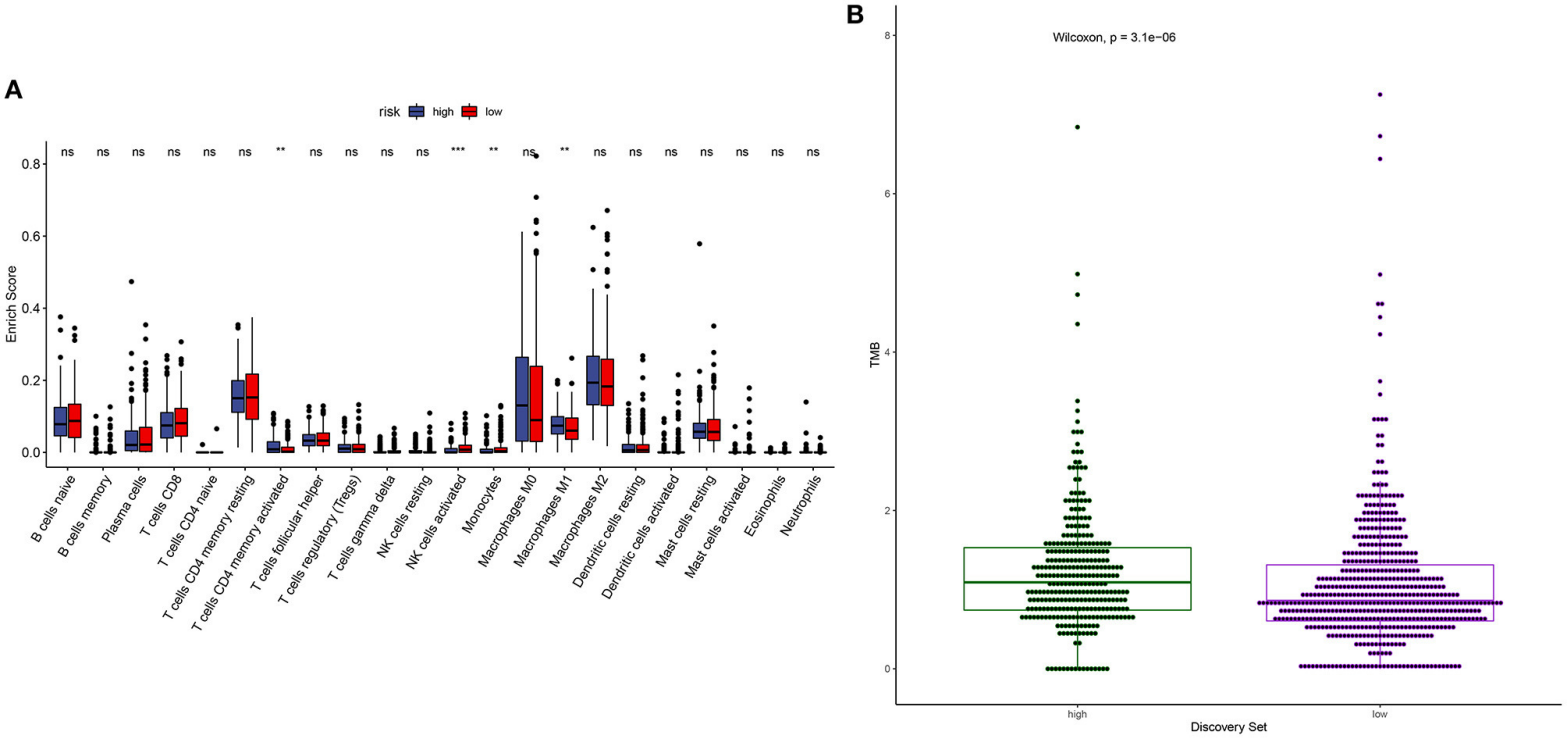
Correlation between YTHDF1 expression and tumor immune microenvironment and response to immunotherapy. **(A)** Box plots for the association between YTHDF1 expression and infiltration levels of immune cells in breast cancer. **(B)** Box plots for the association between YTHDF1 expression and TMB score.

m^6^A modification is closely related to tumor microenvironment cell infiltration in individual tumors (12). Here, we assessed the correlation between YTHDF1 expression and tumor immune microenvironment in breast cancer. Samples with high YTHDF1 expression distinctly exhibited higher infiltration scores of T cells CD4 memory activated and macrophages M1 those with its low expression (Figure 5A; Table 2). Lower infiltration levels of NK cells activated and monocytes were found in subjects with high YTHDF1 expression compared to those with its low expression. Despite the revolutionization of immune checkpoint blockade (ICB) therapy, most patients cannot benefit from ICB therapy (13). We found that high YTHDF1 expression was significantly correlated to higher TMB score, indicating that patients with its up-regulation had a better effect on immunotherapy (Figure 5B). Thus, YTHDF1 expression might be used for predicting the response to immunotherapy.

**Table 2:** The correlations between YTHDF1 expression and tumor-infiltrating immune cells in breast cancer.

**Immune cells**	**Correlation**	***P*-value**
B cells naive	0.040366409	0.386168381
B cells memory	−0.078822212	0.090245572
Plasma cells	−0.037586715	0.41974218
T cells CD8	−0.104667997	0.024304051
T cells CD4 naive	−0.004561896	0.922015306
T cells CD4 memory resting	0.081294778	0.080564543
T cells CD4 memory activated	0.120824713	0.009258723
T cells follicular helper	−0.057847282	0.214088643
T cells regulatory (Tregs)	−0.029972053	0.520014582
T cells gamma delta	−0.039701947	0.394041569
NK cells resting	0.044877857	0.33528083
NK cells activated	−0.218149178	2.15*E*−06
Monocytes	−0.129149449	0.005383934
Macrophages M0	0.031822789	0.49456461
Macrophages M1	0.14172183	0.002237962
Macrophages M2	0.017414151	0.708507533
Dendritic cells resting	−0.028485518	0.540933145
Dendritic cells activated	−0.014160222	0.761216826
Mast cells resting	0.084489829	0.069318397
Mast cells activated	−0.006961211	0.881250387
Eosinophils	−0.056387909	0.225892694
Neutrophils	0.074504463	0.109368677

### Somatic Mutations in High and Low YTHDF1 Breast Cancer

**Figure 6:**
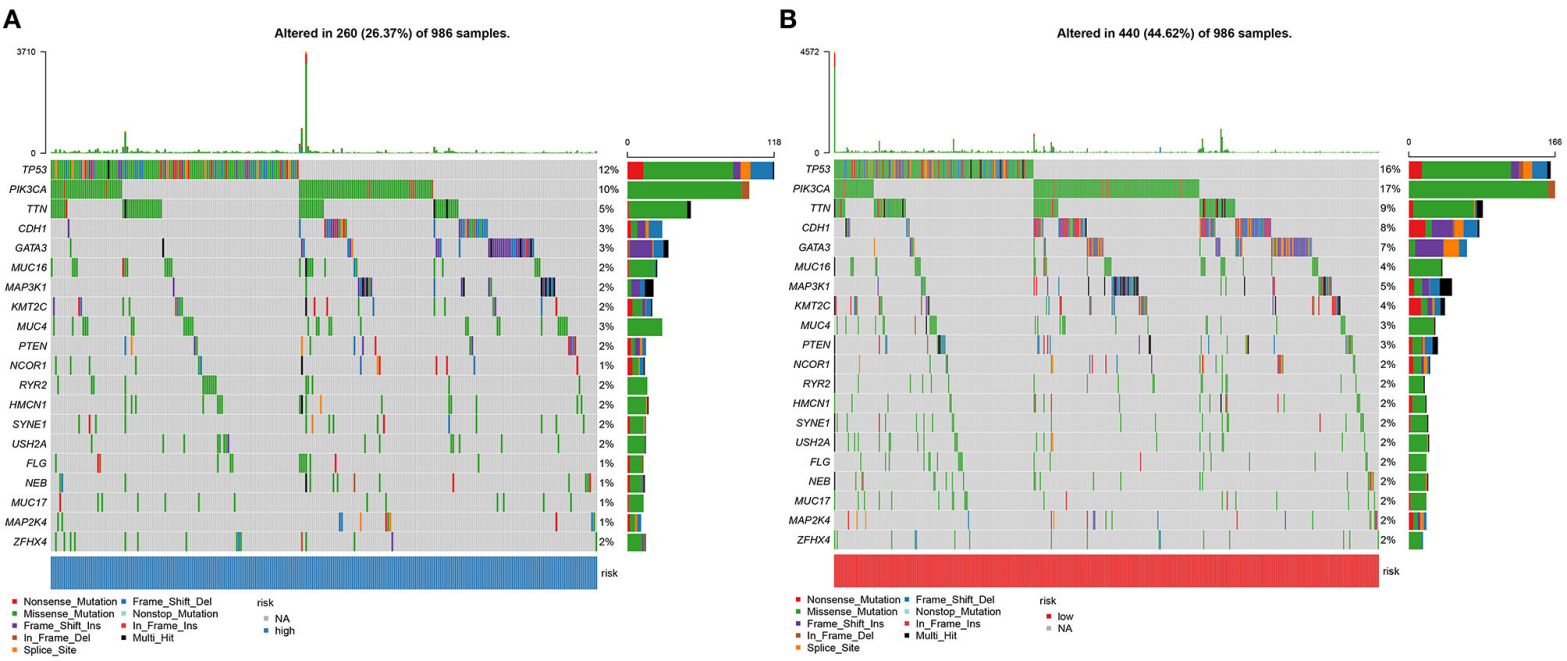
Somatic mutations in high and low YTHDF1 breast cancer. **(A)** The top 20 genes according to mutation frequency in high YTHDF1 expression groups. **(B)** The top 20 genes according to mutation frequency in low expression groups. Each mutation type is identified by a unique color.

Somatic mutations were evaluated in high and low YTHDF1 breast cancer samples from TCGA database. Among 986 samples, 260 (26.37%) occurred somatic mutations in high YTHDF1 expression group. Here, we displayed the top 20 genes according to mutation frequency. As shown in Figure 6A, TP53 (12%) had the most frequently mutated gene, followed by PIK3CA (10%), TTN (5%), CDH1 (3%), and GATA3 (3%). Four hundred and forty samples occurred somatic mutations in low YTHDF1 expression group (Figure 6B). Consistent with high expression group, TP53 (16%), PIK3CA (17%), TTN (9%), CDH1 (8%), and GATA3 (7%) were the top five mutated genes. Both in high and low YTHDF1 expression groups, missense mutation was the most common mutation type.

### Expression Patterns of YTHDF1 in Pan-Cancer

**Figure 7:**
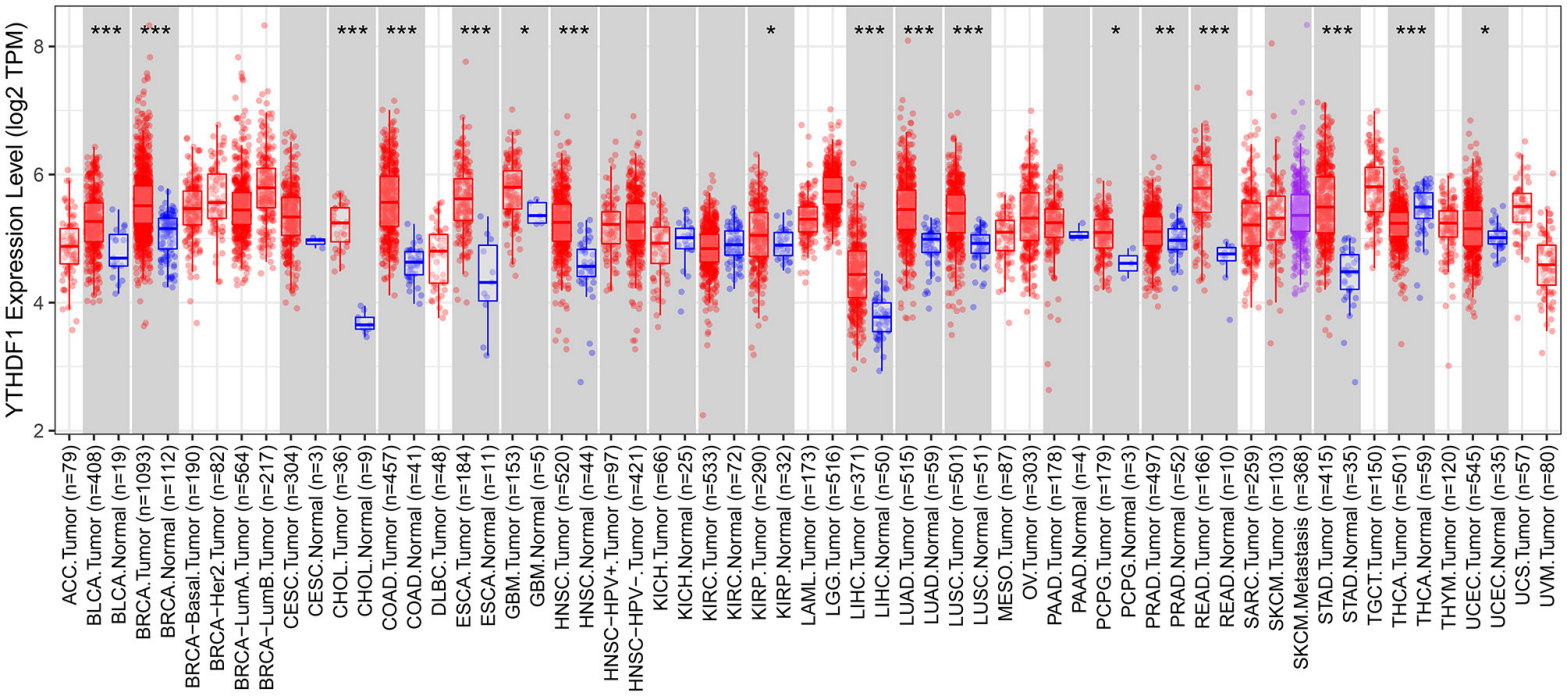
Expression patterns of YTHDF1 in pan-cancer and matched normal tissues. Red indicates tumor specimens and blue indicates normal tissues. Each dot represents one sample. ^*^P < 0.05; ^**^p < 0.01; ^***^p < 0.001.

We comprehensively analyzed the expression of YTHDF1 in pan-cancer and corresponding normal tissues. Up-regulation of YTHDF1 was found in bladder cancer, breast cancer, cholangiocarcinoma, colon adenocarcinoma, esophageal carcinoma, glioblastoma multiforme, head and neck squamous cell carcinoma, kidney renal papillary cell carcinoma, liver hepatocellular carcinoma, lung adenocarcinoma, lung squamous cell carcinoma, pheochromocytoma and paraganglioma, prostate adenocarcinoma, rectum adenocarcinoma, stomach adenocarcinoma, and uterine corpus endometrial carcinoma compared to corresponding normal tissues (Figure 7). However, YTHDF1 was lowly expressed in thyroid carcinoma than normal samples.

### Enrichment Analysis of YTHDF1-Related DEGs in Breast Cancer

**Figure 8:**
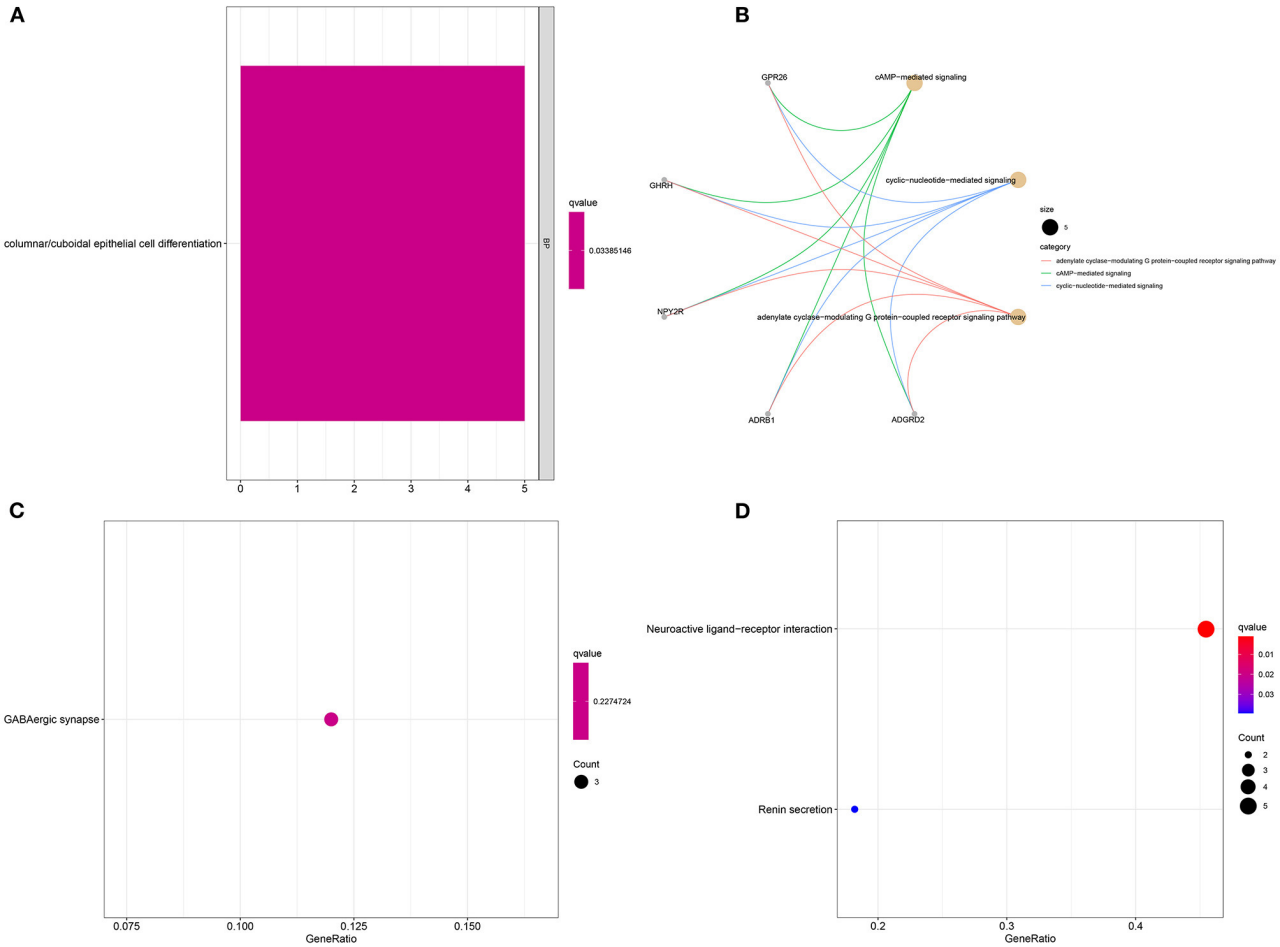
Enrichment analysis of YTHDF1-related DEGs in breast cancer. GO enrichment results by **(A)** up-regulated and **(B)** down-regulated genes. KEGG pathway enrichment results by **(C)** up-regulated, and **(D)** down-regulated genes.

Sixteen up-regulated and down-regulated genes were identified between high and low YTHDF1 expression breast cancer samples (Table 3). Their biological functions were then discovered through GO and KEGG enrichment analyses. We found that up-regulated genes were only enriched in columnar/cuboidal epithelial cell differentiation (Figure 8A). Down-regulated genes were significantly involved in RNA metabolism-related biological processes, such as cAMP-mediated signaling, cyclic-nucleotide-mediated signaling, and adenylate cyclase-modulating G protein-coupled receptor signaling pathway (Figure 8B). Furthermore, they had the molecular functions of G protein-coupled receptor binding and neuropeptide receptor binding. KEGG pathway enrichment analysis revealed that up-regulated genes significantly participated in GABAergic synapse (Figure 8C). In Figure 8D, neuroactive ligand-receptor interaction and renin secretion were distinctly enriched by down-regulated genes.

**Table 3:** DEGs between high and low YTHDF1 expression in breast cancer.

**Gene**	**Low**	**High**	**Log2FC**	***P*-value**	**FDR**
UCP1	0.613741	0.24966	−1.29766	0.000168	0.000431
AC005150.1	0.381289	0.827139	1.117244	4.01E-06	1.38E-05
NDST4	0.153095	0.371715	1.279765	1.01E-06	3.83E-06
LIN28A	0.17983	0.396404	1.140337	5.25E-05	0.000149
SLITRK1	0.120873	0.280966	1.216906	0.000304	0.000742
RHOXF1P1	0.202294	0.553388	1.451838	1.21E-11	9.52E-11
CACNG6	0.383047	0.822432	1.102376	2.79E-05	8.30E-05
SLC4A10	0.249078	0.552009	1.148093	1.74E-06	6.37E-06
TEX19	0.188221	0.450787	1.260016	1.01E-14	1.22E-13
SBK2	0.213431	0.516452	1.274862	7.62E-08	3.46E-07
LINC00844	0.403406	0.198363	−1.02409	2.10E-06	7.57E-06
DPYSL5	0.224008	0.510557	1.188521	0.000552	0.001283
AC025423.3	0.490366	0.98134	1.000895	7.28E-08	3.31E-07
ADGRD2	0.308757	0.142028	−1.12029	0.006414	0.011896
CSMD3	0.101368	0.292825	1.530431	1.46E-09	8.52E-09
AL355075.4	0.287489	0.113742	−1.33774	0.021636	0.035462

### Candidate Therapeutic Agents Against Breast Cancer Based on YTHDF1-Related DEGs

**Figure 9:**
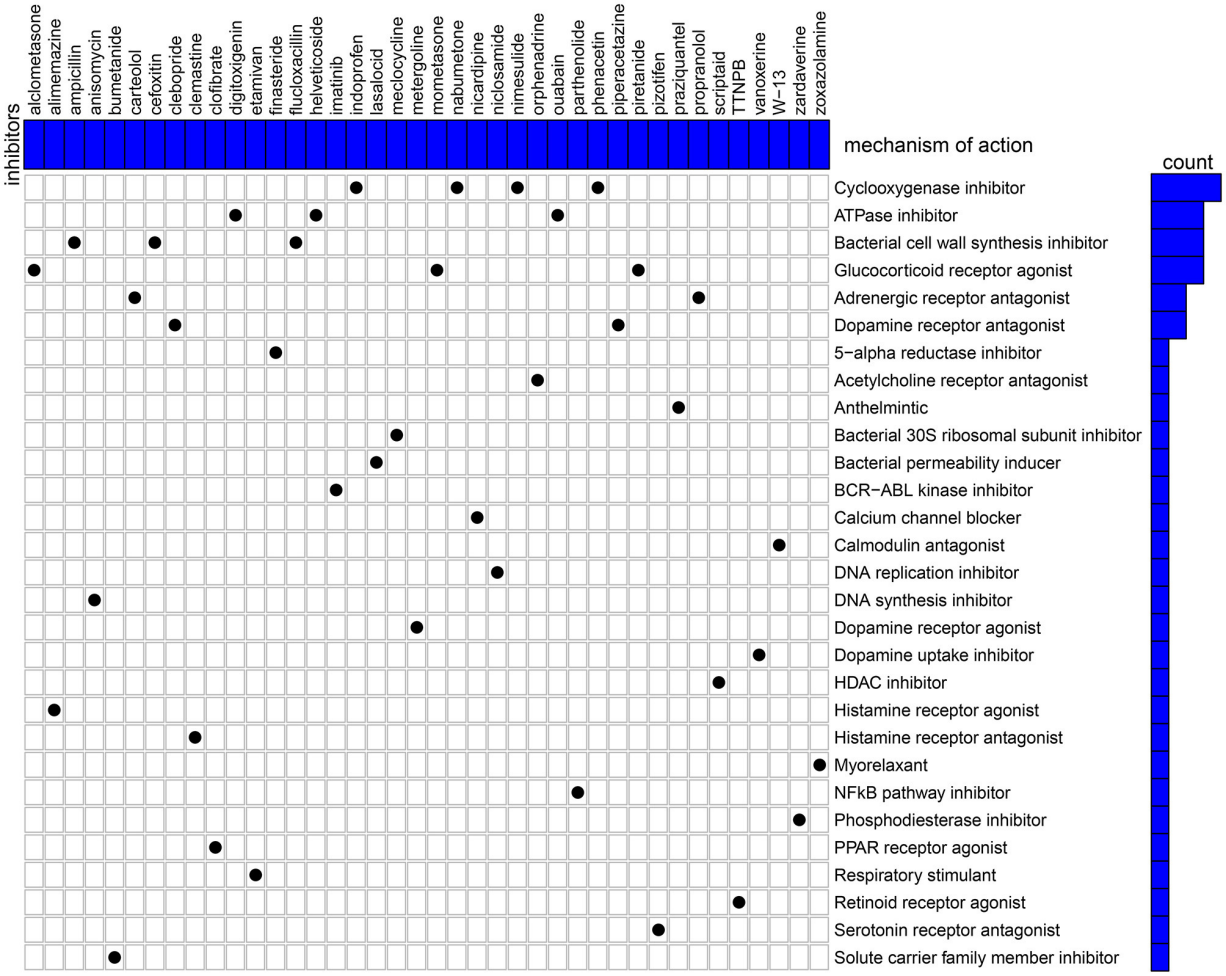
CMap identifies the candidate therapeutic agents related to the DEGs between high and low YTHDF1 expression breast cancer samples. Heatmap shows each inhibitor (perturbagen) and its shared mechanism of action (row).

Candidate therapeutic agents were discovered based on YTHDF1-related DEGs (Table 4). We found that indoprofen, nabumetone, nimesulide, and phenacetin shared the MoA of Cyclooxygenase inhibitor. Digitoxigenin, helveticoside, ouabain shared the MoA of ATPase inhibitor. Alclometasone, mometasone, and piretanide shared the MoA of Glucocorticoid receptor agonist (Figure 9). These compounds could become candidate therapeutic agents against breast cancer.

**Table 4:** Candidate therapeutic agents against breast cancer.

**Rank**	**CMap name**	**Mean**	***N***	**Enrichment**	***P***	**Specificity**	**Percent non-null**
1	Harmalol	−0.82	3	−0.943	0.00028	0	100
2	Flucloxacillin	−0.362	4	−0.808	0.00265	0.0063	50
3	Lasalocid	−0.418	4	−0.807	0.00271	0.037	50
4	Tiletamine	−0.339	4	−0.794	0.00358	0.0057	50
5	Carteolol	−0.26	4	−0.777	0.00511	0.0177	50
6	Ethosuximide	0.371	4	0.767	0.00569	0.0145	50
7	Finasteride	−0.349	6	−0.635	0.00693	0.1402	50
8	Scriptaid	0.507	3	0.818	0.01192	0.1389	66
9	Clemastine	−0.467	3	−0.808	0.01418	0.0504	66
10	Mycophenolic acid	−0.542	3	−0.793	0.01797	0.0974	66
11	Prestwick-857	−0.54	4	−0.696	0.01804	0.0382	75
12	Coralyne	−0.571	4	−0.692	0.0193	0.0067	75
13	Amphotericin B	−0.598	4	−0.687	0.02065	0.0385	75
14	Caffeic acid	0.455	3	0.78	0.02143	0.0208	66
15	Clebopride	−0.582	4	−0.675	0.02487	0.0132	75
16	Nabumetone	0.335	4	0.665	0.02839	0.0325	50
17	Alimemazine	−0.534	4	−0.661	0.0298	0.0272	75
18	(-)-Atenolol	0.376	4	0.658	0.03165	0.0147	50
19	Colecalciferol	0.191	4	0.646	0.03804	0.0469	50
20	Piretanide	−0.392	4	−0.643	0.03909	0.0833	50
21	Zardaverine	0.327	4	0.635	0.04327	0.0725	50
22	Dimenhydrinate	−0.195	4	−0.634	0.04368	0.0432	50
23	Bumetanide	−0.424	4	−0.629	0.04629	0.1622	50

### YTHDF1 Knockdown Restrains Migrated and Invasive Abilities of Breast Cancer

**Figure 10:**
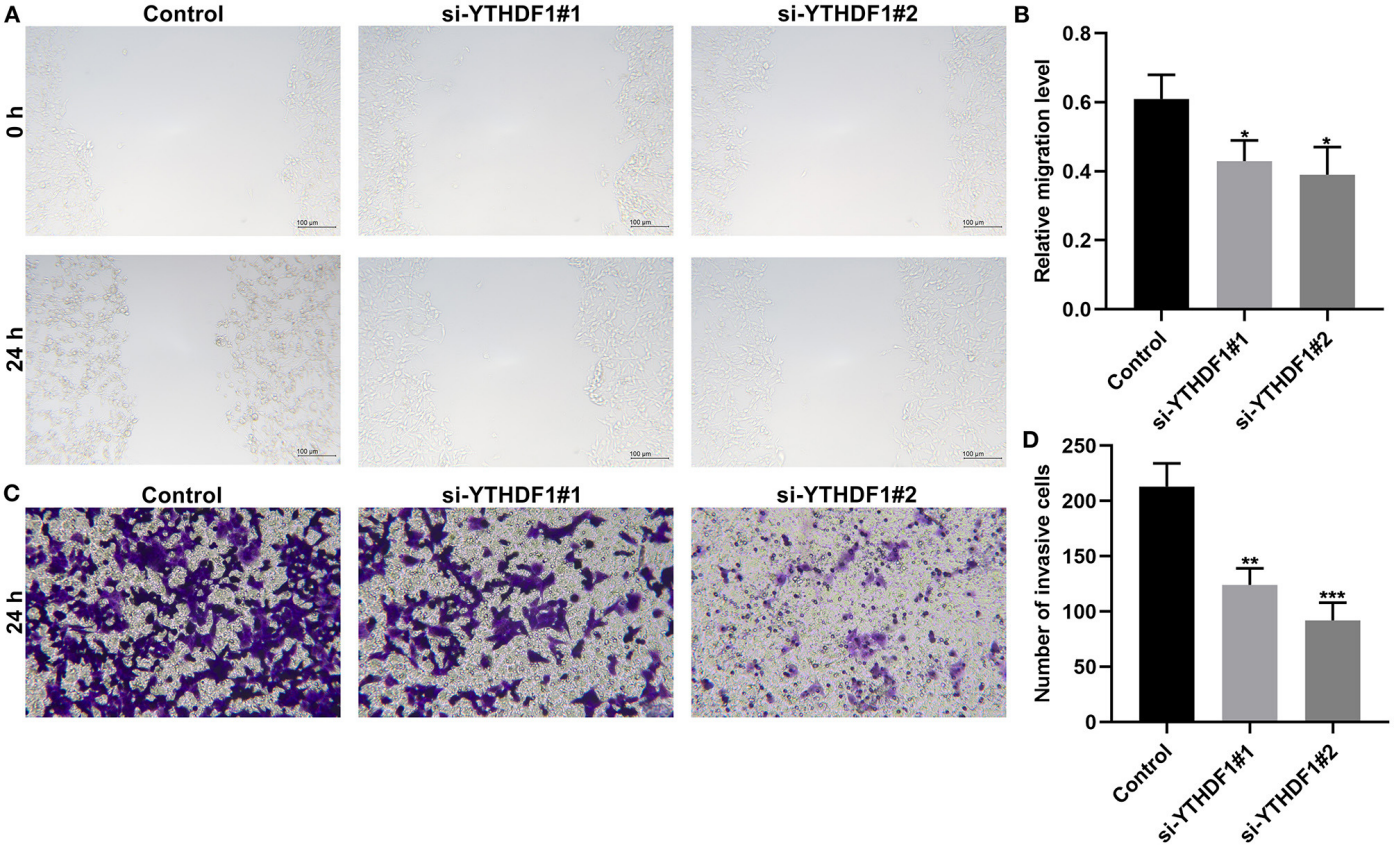
YTHDF1 knockdown restrains migrated and invasive abilities of in MCF-7 cells transfected with si-YTHDF1. **(A,B)** Scratch test for the relative migrated levels of transfected MCF-7 cells. **(C,D)** Transwell for the number of invasive MCF-7 cells. ^*^P < 0.05; ^**^p < 0.01; ^***^p < 0.001.

Following YTHDF1 knockdown, migrated as well as invasive abilities of breast cancer were investigated in breast cancer. The scratch test demonstrated that silencing YTHDF1 markedly lowered the migrated levels of MCF-7 cells (Figures 10A,B). Moreover, the number of invasive cells was decreased by YTHDF1 knockdown (Figures 10C,D).

## Discussion

In this study, we characterized the expression patterns of m^6^A regulators and their implications on survival outcomes, immune microenvironment, response to immunotherapy as well as somatic mutations in breast cancer. Our data highlighted the key roles of m^6^A modification and their regulators in tumor progression and prognosis.

We found that most of m^6^A regulators including METTL14, WTAP, KIAA1429, RBM15, ZC3H13, YTHDC1, YTHDF1, YTHDF2, HNRNPC, and FTO were dysregulated in breast cancer than normal tissues. METTL14 displayed a strong correlation to YTHDC1 and YTHDC2 while YTHDC1 was strongly correlated to YTHDF1 and moderately correlated to HNRNPC. Among them, m^6^A reader YTHDF1 aberration is correlated to undesirable survival outcomes in breast cancer subjects, which exhibited the consistency with the research from Anita et al. (20). The carcinogenic roles of YTHDF1 have been confirmed in previous research. For example, Liu et al. (21) reported that YTHDF1 facilitated ovarian carcinoma progress through controlling EIF3C translation. Bai et al. (22) reported that YTHDF1 accelerated tumorigenicity in colorectal carcinoma. Shi et al. (23) found that YTHDF1 correlated to hypoxia adaptation may contribute to non-small cell lung cancer development. Pi et al. (24) also demonstrated that YTHDF1 promoted gastric carcinogenesis through elevating translation of FZD7. Our pan-cancer analysis revealed that YTHDF1 was up-regulated in most types of cancer. Thus, YTHDF1 could be an oncogene. Our experiments confirmed that silencing YTHDF1 suppressed migrated and invasive capacities of breast cancer cells. The GSEA results demonstrated that high YTHDF1 expression was distinctly correlated to cell cycle, ERBB signaling pathway, oocyte meiosis, pathways in cancer, spliceosome, ubiquitin mediated proteolysis, and WNT signaling pathway. Meanwhile, ribosome was significantly enriched in low YTHDF1 expression group. It has been reported that YTHDF1 could regulate cell cycle progression in hepatocellular carcinoma (25). Recent research has reported that YTHDF1 mediates Wnt pathway activation in intestinal stemness (26). These findings were indicative that YTHDF1 up-regulation could participate in carcinogenesis.

Tumor immune microenvironment affects initiation as well as progress in breast cancer (27). Tumor-infiltrating immune cells are correlated to survival outcomes. Here, we found that YTHDF1 expression was distinctly related to T cells CD4 memory activated, macrophages M1, NK cells activated, and monocytes in breast cancer tissues. Vaccines against dendritic cells have exhibited prolonged survival time in breast cancer patients (28). Tumor-associated macrophages may modulate the efficacy of anti-PD-1/PD-L1 therapy in breast cancer (29). Our data were indicative that YTHDF1 might modulate the immune microenvironment of breast cancer, thereby, affecting tumor progression as well as immunotherapy efficacy. For immunotherapy, the higher the TMB of cancer cells, the increased new antigens may be produced. The higher the immunogenicity of the antigen, the stronger the T cell response, and anti-tumor response, which is more suitable for immunotherapy. Immune-checkpoint inhibitors (ICIs) are novel therapeutic strategies against breast cancer. Nevertheless, only some subjects respond to PD-1 or PD-L1 therapy. As widely accepted, breast cancer patients with high TMB can benefit from immunotherapy (30). Here, we found that TMB score was significantly higher in high YTHDF1 expression group compared to its low expression group, indicating that subjects with high YTHDF1 expression were more likely to benefit from immunotherapy.

The occurrence of tumors is the result of the accumulation of somatic mutations (31). In fact, there are basically non-synonymous mutations in the development of tumors. Because the mutation will increase immunogenicity, but in order to avoid being detected and eliminated by the immune system, tumors often increase immune checkpoints (32). The driver gene mutations can lead to tumors, so that a large number of somatic mutations can produce new antigens, which can activate CD8^+^ cytotoxic T cells, thereby, exerting T cell-mediated anti-tumor effects (33). Therefore, when the number of gene mutations accumulates, more new antigens will be produced, which will be more likely to be recognized by the immune system. Among the 986 breast cancer patients, 26.37% samples in high YTHDF1 expression group and 44.62% samples in low expression group occurred genetic mutations, indicating that the frequency of mutations in breast cancer patients was very high. Among them, missense mutation and non-sense mutation were most frequently. Both in high and low expression of YTHDF1 groups, the five most common mutant genes were TP53, PIK3CA, TTN, CDH1, and GATA3, indicating that these mutated genes contributed to the progression of breast cancer.

Except for breast cancer, up-regulation of YTHDF1 was found in various cancers, indicating that YTHDF1 could be an oncogene. To explore underlying molecular mechanisms of YTHDF1 in breast cancer, we screened DEGs between high and low YTHDF1 expression groups. Our results showed that DEGs were mainly involved in RNA metabolism processes, such as cAMP-mediated signaling, cyclic-nucleotide-mediated signaling, adenylate cyclase-modulating G protein-coupled receptor signaling pathway, second-messenger-mediated signaling as well as adenylate cyclase-activating G protein-coupled receptor signaling pathway. These findings indicated that YTHDF1 promoted tumor progression mainly by m^6^A modification. This study also screened several small molecular inhibitors such as cyclooxygenase inhibitor (indoprofen, nabumetone, nimesulide, and phenacetin), ATPase inhibitor (digitoxigenin, helveticoside, ouabain), glucocorticoid receptor agonist (alclometasone, mometasone, and piretanide), which might be candidate therapeutic agents against breast cancer. More experiments should be carried out to investigate the therapeutic effects of these small molecular inhibitors in breast cancer cells.

## Conclusion

Collectively, this study characterized the dysregulated expression patterns of m^6^A regulators in breast cancer. Among them, YTHDF1 overexpression was distinctly indicative of undesirable survival outcomes. Moreover, YTHDF1 up-regulation exhibited a significant association with cancer-related pathways such as cell cycle, pathways in cancer and Wnt signaling pathway. YTHDF1 expression was significantly correlated to tumor-infiltrating immune cells, indicating that it might contribute to the complexity and diversity of immune microenvironment. Furthermore, subjects with YTHDF1 up-regulation were more likely to benefit from immunotherapy. Several underlying small molecular compounds against breast cancer were discovered based on YTHDF1-related DEGs. In conclusion, our data suggested the implications of m^6^A regulators in survival outcomes, immune microenvironment as well as response to immunotherapy in breast cancer.

## Data Availability Statement

The datasets presented in this study can be found in online repositories. The names of the repository/repositories and accession number(s) can be found in the article/supplementary material.

## Author Contributions

JJ and PT conceived and designed the study. YH, QP, and MW conducted most of the experiments and data analysis and wrote the manuscript. XA, YY, YT, and YJ participated in collecting data and helped to draft the manuscript. All authors reviewed and approved the manuscript.

## Conflict of Interest

The authors declare that the research was conducted in the absence of any commercial or financial relationships that could be construed as a potential conflict of interest.

## Publisher's Note

All claims expressed in this article are solely those of the authors and do not necessarily represent those of their affiliated organizations, or those of the publisher, the editors and the reviewers. Any product that may be evaluated in this article, or claim that may be made by its manufacturer, is not guaranteed or endorsed by the publisher.

## Abbreviations

m^6^A: N^6^-methyladenosine
TCGA: The Cancer Genome Atlas
OS: overall survival
GSEA: gene set enrichment analysis
TMB: tumor mutation burden
HR: hazard ratio
CI: confidence interval
NES: normalized enrichment score
FDR: false discovery rate
DEGs: differentially expressed genes
GO: Gene Ontology
KEGG: Kyoto Encyclopedia of Genes and Genomes
CMap: Connectivity Map.
